# Fiber photometry in neuroscience research: principles, applications, and future directions

**DOI:** 10.1007/s43440-024-00646-w

**Published:** 2024-09-05

**Authors:** Michal Kielbinski, Joanna Bernacka

**Affiliations:** 1 Department of Physiology, Maj Institute of Pharmacology, Polish Academy of Sciences, Krakow, Poland; 2 Present Address: Cancer Neurophysiology Group, Łukasiewicz – PORT, Polish Center for Technology Development, Stabłowicka 147, Wrocław, 54-066 Poland

**Keywords:** Fiber photometry, Calcium imaging, Neurotransmitter detection, Genetically encoded biosensors, In vivo recording

## Abstract

In recent years, fluorescent sensors are enjoying a surge of popularity in the field of neuroscience. Through the development of novel genetically encoded sensors as well as improved methods of detection and analysis, fluorescent sensing has risen as a new major technique in neuroscience alongside molecular, electrophysiological, and imaging methods, opening up new avenues for research. Combined with multiphoton microscopy and fiber photometry, these sensors offer unique advantages in terms of cellular specificity, access to multiple targets – from calcium dynamics to neurotransmitter release to intracellular processes – as well as high capability for in vivo interrogation of neurobiological mechanisms underpinning behavior. Here, we provide a brief overview of the method, present examples of its integration with other tools in recent studies ranging from cellular to systems neuroscience, and discuss some of its principles and limitations, with the aim of introducing new potential users to this rapidly developing and potent technique.

## Introduction

Fluorescent indicator molecules have a long and successful history in the field of neuroscience. Soon after the development of the first practically applicable indicators, such as the calcium sensor *fluo3* [1], calcium-sensing was used to obtain paradigm-shifting results with the discovery of astrocytic calcium waves in vitro [2–4] and calcium dynamics and subcellular patterns in glial cells and neurons [5, 6]. Advances in microscopy techniques soon followed suit, with the first successful multiphoton observations of calcium dynamics in vivo [7]. It is however the development of genetically encoded sensors, based on rapid progress in fluorescent protein engineering [8, 9], that paved the way for this decade’s explosive growth in applications, tools, and experiments utilizing fluorescence to probe various phenomena in the nervous system.

These modern genetically encoded sensors are tailored towards an impressive array of biological targets: metal ions (e.g. Ca^2+^, Zn^2+^, Cu^2+^), second messengers, metabolites, enzymatic activity, pH and redox potential, membrane electrical potential, neurotransmitters, neuropeptides and more [8, 9]. Moreover, because any sensor of this class is simply a genetically engineered protein, it allows the researcher to make full use of advances in targeted gene delivery via viral vectors (including retro- and anterograde vectors allowing for tract-tracing applications) and can be made cell type-specific through either promotor choice or through cell type-restricted recombinase (e.g. Cre) expression.

Probing the fluorescent signal is now also easier than ever, with techniques like fiber photometry (FP), microendoscopes (miniscopes) [10], and multiphoton confocal imaging. For recording in intact animals, FP is by far the most accessible in both cost and complication and thus the most common. Together, these developments offer a powerful toolset for designing fairly sophisticated, spatiotemporally, and anatomically defined measurements of different neural processes.

The basic principle of using FP to perform these measurements is simple enough (see: *‘Fiber photometry: essential principles’*, below). In practice, however, far more nuance is present in choosing, delivering, and testing sensors for FP, and more yet in their practical application in neuroscientific experiments. Specificity, signal processing and analysis, as well as downstream biological interpretation of obtained data all require careful consideration.

For this reason, several in-depth methodological reviews [11, 12], protocols for simple [13, 14] and advanced variants of the method [15, 16], as well as more narrowly aimed domain-specific reviews [17–19] have already been published. This narrative review aims to bridge the gap between these papers by providing an entry point for potentially interested users who are not yet familiar with the technique, but still wondering about how FP could contribute to their research goals. We will use the “worked example” approach, by first providing an overview of fiber photometry and its main moving parts, followed by a discussion of several recent examples of studies in which FP was successfully applied to a variety of research questions at the cellular, system and integrative level, ending with a brief practical guide focusing on limitations and important steps to follow when considering such an experiment, and some thoughts about exciting innovations in the field.

## Fiber photometry: essential principles

Following the development of the first generation of genetically encoded fluorescent calcium sensors [20] and the experimental demonstration of synthetic, small molecule calcium indicator dyes (e.g. BAPTA) for recording cortical activity using two-photon microscopy in vitro and in vivo [7], methods for fast, cheap and easily applicable acquisition of fluorescent signal from awake animals were highly sought after. Progress came in 2005, when a group of researchers from Munich [21] developed and validated in newborn mice a simplified measurement system, consisting of a laser light source, implantable optic fiber, beam splitter cube (for separating the excitation and emitted fluorescent light delivered through this single fiber), photomultiplier detector and amplifier-digitizer (borrowed from a patch clamp setup). Light, delivered from the laser via the implanted fiber, illuminated an area of cortex stained with BAPTA-1, revealing fluorescent light intensity – and thus, calcium concentration – oscillations, readily detectable by the system. Fiber photometry was born.

**Fig. 1:**
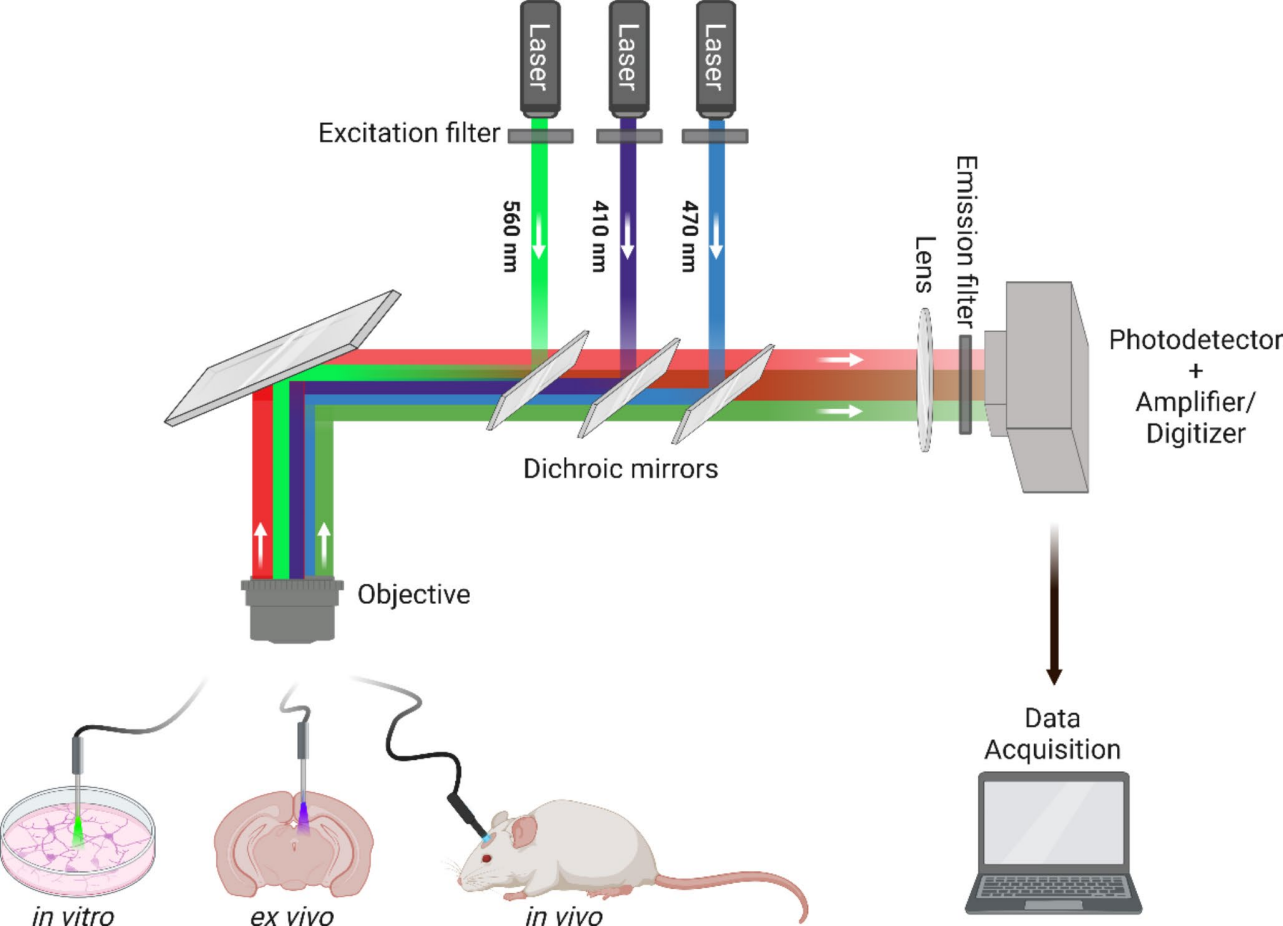
Schematic of a fiber photometry system, in the typical configuration used for monitoring neuronal activity via fluorescence. The system consists of: lasers here depicted at (common) example wavelengths of 410 nm (isosbestic), 470 nm, and 560 nm, with each wavelength used to excite specific sensors, excitation filters selectively passing light of specific wavelengths ensuring precise fluorophore excitation, dichroic mirrors for directing laser light to the objective and separating emitted fluorescence from excitation light for further analysis, objective or implanted fiber focusing excitation light, respectively, onto the sample or into tissue and collecting emitted fluorescence, emission filters allowing only emitted fluorescence to pass while blocking wavelengths corresponding to e.g. excitation or non-specific fluorescence, ensuring signal purity, photodetector with amplifier/digitizer for converting collected fluorescence into an electrical signal which is then amplified and digitized for analysis and data acquisition hardware and software enabling real-time interpretation of data

Over the following decade, the work of several groups brought the technique towards maturity, with vast advances in equipment, measurement strategies, analytical approaches and available methods for control and verification [22–24]. Current approaches range from intensimetric, which is by far the most widespread and commercially available, to much more sophisticated methods using single photon counting to obtain spectral and temporal information beyond simple light intensity. The overall principle, however, remains in most cases similar (Fig. 1). For researchers with a background in image analysis, intensity-based FP readout can essentially be conceptualized as very simplified wide-field fluorescence microscopy, yielding a time series of 1 × 1 “pixel” single “images” (bulk measurements from the fiber’s light cone), and subject to similar fundamental physical constraints – light penetration limited by light scattering and absorption by the tissue and numerical aperture of the fiber [24–26], background autofluorescence, “bleedthrough” in multi-channel recording, etc. The digitized signal bears some familiarity with other electrophysiological or electrochemical signals and can be filtered, processed, and analyzed in a largely similar fashion, offering another entry point for those more familiar with such techniques.

### Fluorescent sensors

Genetically encoded sensors used in FP are a class of engineered fluorescent proteins, based on initial discoveries made in cnidarians, particularly *Aequorea* and *Discosoma* (origins of GFP- and mCherry-like proteins, respectively). In the three decades since their invention, these proteins have undergone an immense amount of development. In the case of sensors, this means that an extraordinary diversity of mechanisms for translating analyte concentrations, molecular events, or other physical phenomena into light have been devised. This includes utilizing Förster energy transfer – FRET (used in many genetically encoded voltage indicators, GEVIs), leveraging various pre-existing properties like pH dependence (e.g. pHluorin), controlled assembly of the fluorophore by apposition of two non-fluorescent parts, or photoconversion/photoswitching of the fluorescent protein. Probably the most common, and most successful, engineering method is the use of circularly-permuted fluorescent proteins (e.g. cpGFP – circularly-permuted Green Fluorescent Protein). In this approach, the C- and N-termini of a fluorescent protein are fused with a short peptide linker, and a new “opening” is created, which can be then modified with a sensing domain designed to bind to a given analyte and, in response, change the conformation of the engineered chromophore. The structure of the sensor protein, particularly the linker sequence, is optimized so that upon ligand binding the fluorophore increases its fluorescence by orders of magnitude (for an in-depth review, see: 9).

The prototypical sensor in this class is the famous genetically-encoded calcium indicator (GECI) family, GCaMP [27], in which calmodulin (CaM) and myosin light chain kinase sequence (M13) form the calcium-binding sensing domain and a cpGFP molecule forms the reporter domain. The GCaMP family has undergone many rounds of refinement and optimization to achieve increased sensitivity and specificity, superior kinetics, improved dynamic range, and even different spectral properties (yielding different-colored sensors). This is possible thanks to improvements in mutagenesis, screening, and *in silico* modeling of the molecule. Rationally designed sensors for neurotransmitters and neuromodulators are now readily engineered, utilizing ligand binding domains obtained from various biological systems, most commonly mammalian G protein-coupled receptors (GPCRs), used e.g. in the dLight and GRAB (G Protein-Coupled Receptor Activation-Based) sensor families [28, 29], and microbial periplasmic binding proteins (PBPs), used in the SnFR sensor family (e.g. iGluSnFR – intensified Glutamate Sniffer Fluorescent Reporter) [30, 31]. This approach allows for highly modular “building blocks’’ to be used in the construction of new or improved tools: for instance, in a recent study [32], the authors developed and optimized a suite of sensors based on known GRAB linker and chromophore sequences for several neuropeptide molecules, with sensing domains made from their cognate receptors.

Unsurprisingly, a wide array of sensors for various neurotransmitters, neuromodulators, small molecules, and other analytes has been developed by research groups worldwide. While likely non-exhaustive (for practical reasons), curated databases of fluorescent biosensors are available e.g. at the Addgene repository [33] or the University of California, San Diego Fluorescent Biosensor Database [34] available publicly at: *biosensordb.ucsd.edu*.

### What questions can photometry answer?

The ability to detect events like calcium elevations, neurotransmitter release, or changes in membrane voltage is, of course, very powerful and widely applicable to a variety of research questions at different scales. On the cellular level, the timing of neurotransmitter uptake and release events can be studied (in vitro or ex vivo). On the structure or ensemble level, shifts in activity in a given structure, or within a specified neuronal population in the area, or the activity of specific inputs (e.g. glutamatergic, dopaminergic) can be observed. In combination with other techniques, in particular optogenetic stimulation, FP can be leveraged for tract-tracing experiments. It can be readily combined with behavioral observation in freely moving animals, where photometry compares favorably with other methods routinely used for neurotransmitter detection, e.g. microdialysis or cyclic voltammetry. The technique can be adapted for recording both fast (milisecond) and longer (minutes to hours) events; in addition to the advantage of the measurement being performed at the cell expressing the sensor, in comparison with a fairly large electrode or probe sampling diffuse analyte in the tissue (for highly detailed comparison with these techniques, see: [35, 36]). Depending on available equipment, multiplex measurements (two or more concurrent channels from a single site, multisite recording, or both) are possible. Together, those capabilities make FP a powerful tool for studying the contributions of various systems, brain areas, circuits, inputs, or cell types to behaviors of interest.

A typical experiment utilizing FP for in vivo recording consists of at least the following steps:

- Preparation: viral transduction, where the vector carrying the sensor of choice is stereotaxically introduced into the structure of interest, and optic fiber implantation; these two steps are typically performed together during a single surgery to save time and minimize the burden on the animal, although performing fiber implantation at a later date can allow for optimizing fiber placement by performing intraoperative measurements;
- Data acquisition (including recording the experimental signal as well as appropriate controls, obtained either concurrently or in sequence to verify the specificity of the observed fluorescence, as well as correlating fluorescent signal together with any experimentally-relevant events – behavioral, electrophysiological, etc.);
- Data pre-processing, in which raw signal is typically, in sequence: filtered (usually with a low-pass filter removing high-frequency electrical noise from the typically millisecond-scale FP signal), corrected for slow baseline drift (predominantly loss of fluorescence due to bleaching), corrected for the more stochastic artifacts from animal movement, tissue and optic path shifts, physiological changes in parameters like local pH, blood flow and oxygenation (e.g. by using a constitutively active reference channel, or by exciting the fluorophore at its isosbestic point, i.e. the wavelength where fluorescent emission is theoretically independent of analyte binding) and finally normalized, most often by expressing the obtained fluorescence as ΔF/F (signal over background) or z-score;
- Analysis, which typically consists of visualizing the data with e.g. peri-event histograms of fluorescent activity corresponding to some experimentally meaningful signal (cue presentation, stimulation, performing an operant task) and, in that window, obtaining simple measures (peak amplitude, latency, area under the curve) or attempting to model signal intensity or kinetics using linear or non-linear methods, with observables or manipulations (cues, tasks, stimulation) as predictors; alternatively, the signal can be analyzed “wholesale”, with no a priori event windows.

Like any technique, however, FP is not free from serious limitations. These come in two essential flavors: fundamental limits of the technique itself and technical difficulties which need to be taken into consideration. Key among the former is the issue of quantification. For a variety of other methods, such as electrochemical neurotransmitter detection, the relationship between analyte concentration and signal amplitude is known a priori and typically linear, although often influenced by confounding factors like the presence of chemically similar analytes or electrode fouling (which presents a hard, but often tractable, methodological challenge). This makes calibration, and therefore obtaining absolute concentration values, essentially possible [37, 38]. In contrast, for photometric measurements, interpreting fluorescence intensity in terms of neurotransmitter or ion concentration is not so straightforward. Even if the efficacy and lifetime of fluorescence emitted by the sensor in the presence of known concentrations of the analyte is known from in vitro experiments, this knowledge is in no way sufficient for determining this relationship for an implanted optic fiber collecting fluorescent light in situ. Fluorescence intensity or photon count is always a product of the unknown and essentially unknowable properties of the tissue microenvironment and moment-to-moment parameters – e.g. expression pattern and density, physical state (with phenomena like quenching or bleaching), three-dimensional location within the light cone – of the fluorescent puncta (active sensors) from which light is collected in any given frame.

This fundamental limitation is a well-known challenge for which some approaches, both current and in development, may provide potential solutions (see: *‘Photometry in the near future’*). However, in the near term, it largely limits the ability of FP to meaningfully measure processes in the “raw” intensity or amplitude domain, or quantitatively compare signals and measurements. Consequently, FP experimental design is focused on obtaining information about kinetic properties of the signal, relative changes in fluorescence, and reliable co-detection of other inputs (behavioral, electrophysiological, etc.) and fluorescent signals from a specific target, with sufficient fidelity. These considerations dictate sensor choice, experimental procedures, and analytical approach, from signal pre-processing to interpretation of obtained data.

### Photometry in current research

Despite their limitations, fluorescent sensors are more than capable of generating valuable insights. At the time of writing, the general phrase “fiber photometry” returns almost 700 records in the PubMed database. Most (600/700) of this rapidly developing field consists of studies published in the last five years. Any sort of systematic review of this literature would thus be already out of date by the time of this article’s publication. Therefore, what follows is a subjective collection of example papers illustrating how research questions at different levels – from cellular to systemic – can benefit from incorporating photometric techniques.

### Cellular processes visualized with photometry

An early example of clever application of fluorescent sensors to the study of neurotransmitter signaling dynamics comes from Condon et al. [39]. The authors set out to measure the kinetics of dendrodendritic dopamine release and modulation of dopaminergic neurons in the substantia nigra via D2-like autoreceptors. Combining fluorescent measurements made using dLight1.3b – a dopamine sensor – with photoactivation via optic uncaging of a modified analog of sulpiride (a D2-like receptor antagonist), they performed patch clamp and microscopic recordings ex vivo. This allowed them to decipher the relative sequence, timing and critical periods which determine amplitude and duration of inhibitory G protein-coupled inwardly rectifying potassium channel currents, by tightly controlling local sulpiride uncaging. In addition to its primary findings, the paper offers some additional insights for researchers focused on catecholamine neurotransmitters, e.g. indirect comparisons of dopamine and noradrenaline effects on D2-like receptors, or evidence for relative competition for neurotransmitter binding between native GPCRs and dLight (in essence, an artificially introduced GPCR).

A similar example, where electrophysiological, imaging, and photometric methods were combined to probe plasticity in hippocampal neurons, comes from a study by Badia-Soteras et al. [40]. Here, the authors were interested in hippocampal plasticity related to fear conditioning. First, they demonstrated, using electron microscopy, that fear learning elicits relatively fast (~ 30 min) retraction of astrocytic endfeet from synaptic clefts in the CA1 area. They then developed a CRISPR-based construct for selective partial ablation of ezrin – a key astrocytic cytoskeletal protein associated with fine processes – to elicit a similar effect in vivo in a controlled fashion. This was followed by a series of experiments combining whole-cell patch clamp and two-photon imaging using iGluSnFR, in which the authors found that ezrin ablation and astrocytic endfeet retraction reduces glutamate availability in the synaptic cleft, increases spillover, enhances extrasynaptic NMDA receptor activation and results in slower decay kinetics (in other words, slower uptake) of glutamate. Finally, they demonstrated that ezrin ablation in CA1 results in increased expression of recently acquired fear memory. These results together suggest a novel glial mechanism involved in processing aversive memories.

Stepping momentarily away from detecting neurotransmitters, in another early proof of concept study, Mei and coworkers [41] used in vivo FP for tracking gene expression temporal patterns in defined structures and neuronal populations. They achieved this by constructing a reporter based on Venus (a protein from the GFP family), driven by the promoters of Cry1 and Per2 – essential circadian clock genes. With this, they were able to successfully record transcriptional circadian rhythms in both the suprachiasmatic nucleus (SCN) and the hippocampus, as well as a defined cell population of vasoactive intestinal peptide-expressing SCN neurons (by using VIP-Cre mice). Since reporter constructs based on either Venus or other fluorescent proteins have been developed for many genes of interest, such as the widely used immediate-early neuronal activity and plasticity marker cFos [42], FP opens an interesting avenue for selective in vivo recording of transcriptional activity in behaving animals – provided that some of these molecular tools can be repurposed and refined for this application.

### Ensemble-level insights from photometry

Historically, as GCaMP was the first widely available genetically encoded fluorescent sensor, observing neuronal activity patterns at the levels of structures or ensembles of cells has always been at the forefront. This remains an important use case for GEVI and GECI sensors; however, the fundamental limitation outlined above – that of indirect measurement – fully applies. Much work is being done to describe in more detail how fluorescent signal recorded from these sensors translates to excitatory and inhibitory transmission [43], and to systematically assess if and when parallels between e.g. calcium fluctuations and spiking activity can be drawn [44]. Those questions are, however, far from solved, and it is unlikely that sensors combined with FP or microscopy would replace more direct, electrophysiological (extracellular and patch clamp) techniques. They, however, offer two very important advantages that make them excellent extensions of the neurophysiological toolbox: the ability to interrogate processes and cellular targets not easily accessible via electrophysiology, and to selectively target sensor expression in a molecularly defined fashion.

A great example of the former is a study by Tan et al. [45]. Here, the authors used fluorescent sensors in combination with optogenetics to probe astrocyte and vascular function. They asked whether changes in astroglial calcium signaling pH are correlated with increased theta band activity in the lateral habenula (LHb) – a phenomenon strongly associated with aversive emotional experiences, fear and anxiety. Their experimental workflow combined a custom-made multi-channel FP apparatus with a number of molecular tools: a viral construct carrying an albumin-mScarlet fusion protein expressed in the liver and shed subsequently into plasma, allowing for local fluorescent measurement of brain blood volume [46], a ratiometric FRET-based calcium sensor YC_nano50_ (a member of the *cameleon* ratiometric sensor family), a pH sensor E^2^GFP and a transmembrane proton pump, ArchT – the latter three expressed under the control of the Mlc1 promoter, which is active in habenular astrocytes. With these tools, they observed temporal patterns of Ca^2+^ and pH in mice both during physiological situations, such as sleep and wake, and in anxiogenic conditions. Upon transition to an anxiogenic environment, they observed an (expected) lasting increase in LHb theta band activity in mice, which was accompanied by increased local blood perfusion. Conversely, astrocytic calcium concentration exhibited a sharp peak upon transition, followed by a sustained decrease. Similarly, astrocytic pH was also decreased while the animal was in the stressful environment. Further, the authors hypothesized that counteracting these pH changes would elicit an anxiolytic effect. Their expectation was substantiated, as optogenetic activation of ArchT (driving proton outflow from the cell) attenuated the anxiety-associated increase in habenular theta power, while increasing the time spent by the mice in the brightly lit compartment in a light-dark box test. Taken together, these results seem to corroborate the claim that astrocytes play an important role in modulating theta band oscillations in LHb – and, therefore, anxiety and aversion [47, 48].

Meanwhile, in two recent excellent papers [49, 50], other authors made full use of genetically-defined selective cellular expression of sensor proteins. This research builds upon previous studies [51, 52] where intersectional genetic tools and single-cell transcriptomics were used to identify and describe molecular subtypes of dopaminergic midbrain neurons with distinct physiological and anatomical properties. In the first study [49], Azcorra and coworkers used GCaMP6f, selectively expressed in subsets of dopaminergic neurons of substantia nigra *pars compacta* defined by their expression of Calb1, Vglut2 or Anxa1. They determined the innervation distribution in the dorsal striatum arising from these neurons, and recorded their activity in response to locomotion (acceleration and deceleration on a treadmill), as well as their responses to aversive and appetitive stimuli. They found distinct subtype-specific response patterns to acceleration and deceleration, with preference for the former in Anxa1 + neurons, and for the latter in Calb1 + and Vglut2 + neurons. Moreover, Calb1 + and Vglut2 + axonal responses to salient stimuli were biased for appetitive and aversive stimuli, respectively, while Anxa1 + neurons in general exhibited very little activity in response to these events. In freely moving animals, calcium responses were in general strikingly cross-correlated in neurons from a particular subtype; this finding corroborates the now emerging view that various functions typically ascribed to dopaminergic neurons (valence, reward prediction, driving locator activity, etc.) are subserved by partially overlapping, but functionally heterogeneous neuronal populations, and not simply “dopamine neurons” *sensu lato*. The second study, by Avvisati et al. [50], combined extracellular recording in the midbrain with FP in the striatum and nucleus accumbens in molecularly defined neurons during Pavlovian learning. Here, too, the authors describe a partially overlapping, but spatiotemporally and molecularly distinct pattern of responses to specific components of the behavioral task: predictive cue, reward presentation, consumption, and non-rewarded spontaneous movement, noting also a high degree of cross-correlation in activity between axonal and somatic compartments. They argue against strict axial or orthotopic organization of these inputs, and for clustered gradient organization into hotspots of functionally similar dopamine neurons. Such organization, would help explain why, over several decades of research on the dopamine system, various groups reported findings that were often internally consistent, but difficult to reconcile. It is also worth noting that these two studies are, to a large degree, compatible and present relatively similar conclusions, despite coming at the problem from opposite sides: the former is primarily FP-based and focused on dopaminergic terminal areas in the striatum, the latter is primarily electrophysiological and focused on the somatic compartments in the midbrain, with FP being a secondary technique.

### Systems neuroscience and beyond

The area in which photometric techniques show perhaps the most immediate promise is the study of defined neuronal circuits in the context of complex animal behavior. Here, again, the ability to probe both neuronal activity and transmitter release in circuits defined by projections or molecular markers, often in remote areas of the brain, at different timescales and over a long time, offers some advantages over electrophysiological and electrochemical methods.

An example of this type of experiment comes from Tong et al. [53]. The authors sought to test the hypothesis that altered activity in neuronal projections from the medial prefrontal cortex (mPFC) to the lateral habenula contributes to the development of anxiety- and depression-like states in response to chronic stress. The study made heavy use of retrograde monosynaptic tracing using recombinant adeno-associated virus vectors (rAAV2-Cre) microinjected into the LHb, for cell labeling and driving the expression of GCaMP7s as well as chemogenetic effectors hM3Di and hM4Di in mPFC. They were able to determine that increasing (with hM3Di) or decreasing (with hM4Di) the activity on LHb-projecting mPFC neurons respectively enhanced or alleviated depressive- and anxiety-like behavior in a battery of tests. They then recorded calcium signals from LHb-projecting cortical neurons located in the dorsomedial and ventromedial PFC (roughly equivalent to prelimbic and infralimbic cortex) as well as the anterior cingulate cortex. They found increased activity in response to stressful stimuli; conversely, sucrose consumption inhibited this circuitry. The magnitude of the observed effect was maximal in the dorsomedial part, prompting the authors to then return and manipulate mPFC-LHb circuitry in the ventromedial, dorsomedial, and cingulate cortex separately (by confining viral spread to a smaller, specific area). With that, they confirmed that manipulation of prelimbic (dorsomedial) mPFC, as opposed to the other two areas, is sufficient for modulating anxiety- or depression-like behavior. This work thus provides a powerful demonstration of anatomically-defined, circuit-specific measurement and manipulation.

In a similar vein, another group of authors sought to elucidate the previously undefined role of glutamatergic projections from the thalamic intralaminar nucleus (ILN) into the dorsal striatum in action selection in mice [54]. They used a similar approach, injecting a retrograde viral vector into the striatum to identify thalamic neurons in the ILN for patch-clamp recording and to drive GCaMp6s expression in striatum-projecting ILN cells. They then trained mice in an operant task (lever pressing for sweet pellet reward, fixed ratio progressing from FR1 to FR5, followed by FR5 with a time limit) and used FP to assess how activity in this pathway corresponds to different aspects of this behavior. Activity in the ILN-striatum pathway was always correlated to action initiation. As the animal learned the task, subsequent presses elicited smaller increases in its activation. In addition, when the animal was successfully conditioned – in the intermediate and late trials – lever extension (signaling reward availability) also elicited a robust increase in activity. Finally, the magnitude of calcium response was negatively correlated to response latency and much larger in successful versus unsuccessful trials. Interestingly, these findings were not replicated in a Pavlovian paradigm, nor were they abolished in response to reward devaluation or differed between rewarded and unrewarded (but successfully completed) trials in extinction sessions.

Finally, in a recent study by Amo et al. [55], the authors used several fluorescent neurotransmitter sensors in combination to determine the activity of glutamatergic inputs onto ventral tegmental area (VTA) dopamine neurons (using a glutamate sensor SF-iGluSnFR), calcium transients in the somatic compartment in the VTA (with GCaMP7f), and dopamine release in the ventral striatum (with GRAB_DA2_) during simple as well as sequential Pavlovian conditioning and exposure to aversive stimuli. They aimed to elucidate how glutamatergic inputs and excitation-inhibition balance in the VTA in general shape reward prediction error computation. They first found an expected relationship between predictors and glutamatergic inputs driving dopamine release: namely, both signals scaled directly with reward probability in simple conditioning, and both glutamate and dopamine transients corresponding to the proximal cue in sequence were scaled inversely by reward probability of the distal cue. Then, the authors observed that reward omission is encoded by inhibition of the glutamate input. However, somewhat surprisingly, aversive stimuli were found to be accompanied by robust glutamatergic activity that was presumably only counteracted by GABAergic inhibitory inputs onto dopaminergic neurons (which, as a result, paused in response to aversive stimuli). As a final step, the authors show that opioid administration shifts the inhibitory-excitatory balance on dopamine neurons, attenuating the GABAergic input and reducing the post-stimulus pause in both intracellular calcium in dopamine neurons and dopamine release. Together, these data, if corroborated, would suggest a revision of some long-standing hypotheses about reward calculation in dopamine neurons as well as the relative roles of glutamate and GABA in encoding salient events.

As the above examples illustrate, FP along with other photonic techniques is widely applicable across different domains of neuroscience, making it an increasingly attractive and popular tool. In the following section, we will discuss some of the methodological intricacies that may come into play when designing similar experiments.

### Photometry in practice

**Fig. 2:**
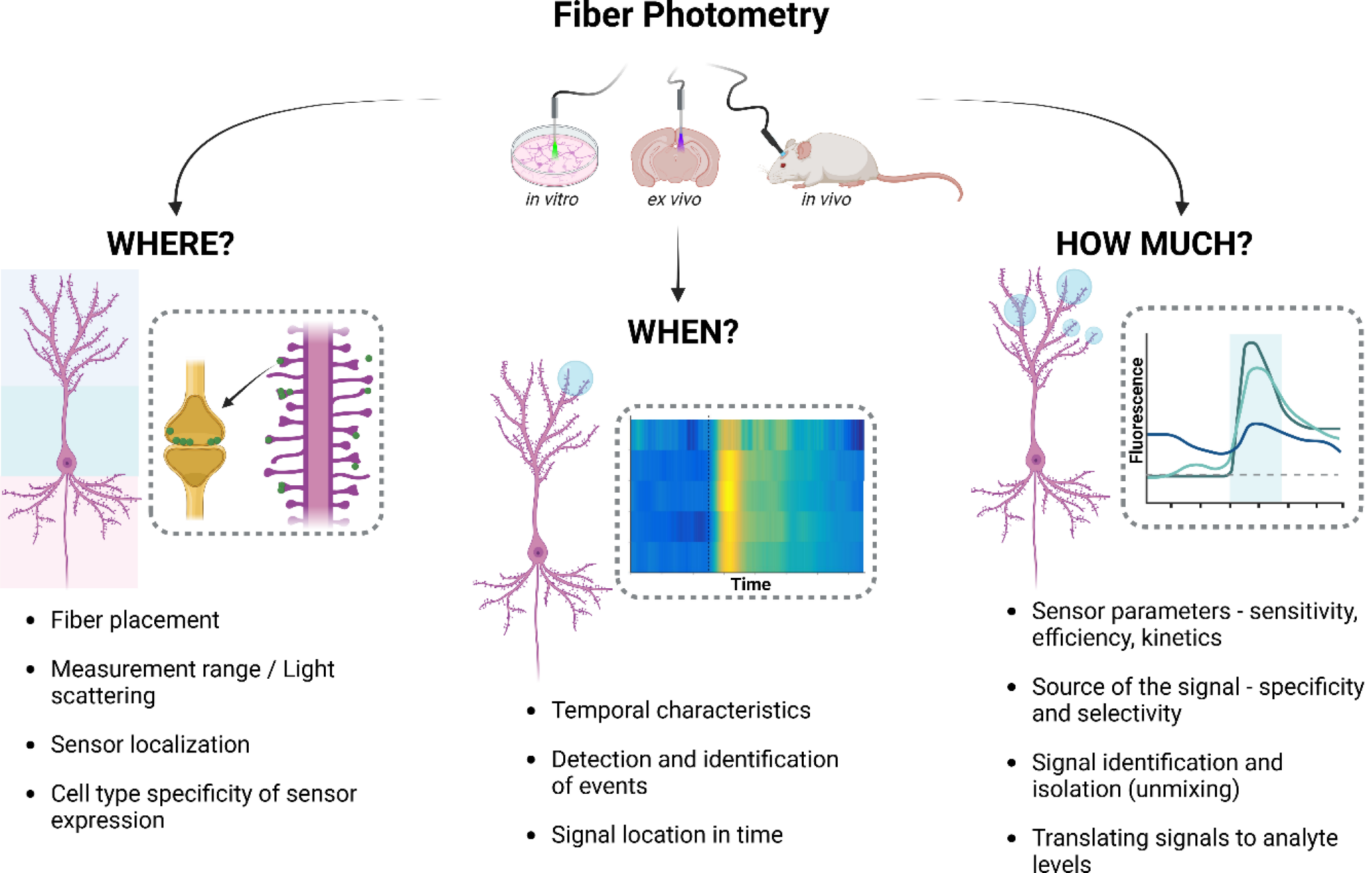
Key aspects of fiber photometry, categorized into three main questions: “Where?”, “When?”, and “How much?” fluorescent signal is detected. A typical FP experiment can be broken down to a combination of those broad classes of questions, which remain relevant at all stages of the experiment – from sensor choice and study design, to experimental protocol, to verification and data analysis

Detailed technical protocols for performing FP are readily available both for the most commonly used intensity-based [13, 14] and for more complicated, but more powerful photon counting variant [15]; for this reason we will refrain from describing the surgery and data acquisition step by step, instead presenting some general considerations and caveats that should be kept in mind when conceptualizing, planning and carrying out FP experiments (Fig. 2), for the most part focusing on basic intensimetric measurements, as these represent the most common and widely available approach.

### Experimental design and sensor choice

As with any experiment, proper planning is key to obtaining reliable and reproducible results. The first step is to determine the type of study that will best answer the research question, considering options such as in vitro, ex vivo, and in vivo studies. For in vivo recordings, it is necessary to decide whether they will be conducted in anesthetized or freely moving animals. If freely moving animals are used, careful consideration of the specific behavioral paradigm is necessary: e.g. how the behavior will be quantified (are there discrete events, what explanatory variables will be generated), is the planned behavioral procedure practical in tethered animals.

Another key aspect of planning is deciding how the signal of interest will be elicited. Neurotransmitter release or the activity of a given circuit can be triggered by electrical, optogenetic or chemogenetic stimulation. Alternatively, one might focus on a specific action (e.g. lever press), or response to a stimulus, such as stress or reward presentation. The chosen experimental paradigm will influence the next critical planning step: the selection of appropriate sensors. For in vitro and ex vivo experiments, high-brightness and stable sensors are ideal due to controlled conditions and ease of monitoring. On the other hand, in in vivo experiments, especially with freely moving animals, robust and photostable sensors that can handle movement artifacts are preferred. Additionally, an important aspect of this type of experimentation is the high sensitivity of the sensors, which is crucial for studying subtle changes. However, this high sensitivity is often associated with slower sensor kinetics. Furthermore, in certain conditions, the dynamic range (the difference between minimal detectable and maximal signal) may become a constraining factor, as with high levels of analyte the response could be saturated. An optimally chosen sensor, therefore, would operate between the physiological “ceiling” and “floor” to generate useful signals. As an example, measuring dopamine, which varies wildly in concentration between striatum and mPFC, would probably benefit from tailoring sensor choice to the specific structure of interest.

As mentioned above, the type of stimulation also affects the choice of sensor. For electrical stimulation, the optimal sensors are those with exceptionally fast kinetics, as they are essential for detecting rapid changes in neuronal activity or neurotransmitter release. However, such sensors often demonstrate lower sensitivity and selectivity. On the other hand, when coupling FP with optogenetics, it is crucial to select sensors that avoid cross-talk and ensure that the sensor’s fluorescence is not affected by the light used for optogenetic manipulation. Similarly, for chemogenetic manipulation, often used in long-term behavioral studies, sensor choice should be compatible with the requirements of the technique: here, sensor stability over time becomes the primary concern. These sensors typically have slower response times, prioritizing long-term stability over fast kinetics.

In the context of sensor specificity, especially in the case of GPCR-based sensors, cross-activation by molecules with a similar structure can be expected, as occurs physiologically, e.g., with noradrenaline acting on dopaminergic receptors [56]. Moreover, like their native GPCRs, sensors can bind to synthetic ligands. This means that the compatibility of a given sensor should be taken into account, e.g. with the pharmacological treatment being tested. It is at this point also that any confounding effects of the sensor itself on neurotransmission should be considered – that is, the researcher should decide whether they would impact the question at hand. These are subtle phenomena like competition for ligand binding between native GPCRs and sensors [39], or “partial” – low amplitude – intracellular signaling events that may arise from GPCR-based sensors in which the binding domain retains a larger part of the original sequence; this has been observed in some GRAB family sensors [17, 57]. If those phenomena are likely to affect the planned measurements of e.g. second messengers or synaptic dynamics, they should be accounted for via appropriate controls.

Regardless of the type of experiment, in any paradigm in which more than one sensor is to be used together, it is extremely important that the sensors used simultaneously are excited by light of a different wavelength. Since no optical component (such as dichroic or filter) is perfect, and since excitation spectra of various fluorophores often overlap – as seen in a frequent scenario of combining a green and a red fluorophore, in which case the tail end of the spectrum of the more redshifted molecule usually extends over the excitation peak of the green molecule – one should keep in mind the spectral properties of both the sensors and the hardware. Using sensors of similar brightness and at similar expression levels makes limiting signal cross-contamination easier, versus a situation where one fluorescent indicator is significantly brighter, or expressed in much higher quantities, than the other. In all cases, even when signals are acquired sequentially, close examination of traces from both channels is warranted: after background correction, minimal crossover signal should be observed.

Another crucial aspect of planning FP experiments involves selecting an appropriate control. This ensures the reliability of results by managing signals arising from factors such as animal movements, environmental noise or incident light, tissue autofluorescence and local conditions – blood flow, oxygenation, local pH, as well as controlling how the signal changes over time due to bleaching, which is especially important for long recordings. Various control signals can be employed when measuring fluorescence intensity, with one common option, found in many commercially available systems, being isosbestic signal. This signal should not, in theory, be influenced by experimental conditions, including excitation or emitted light from the sensors used, as well as fluctuations in neurotransmitter levels or neuronal activity. There are alternative solutions as well, including constitutively fluorescent protein expression (e.g. mCherry control for a sensor combined with GFP), mutant ligand-insensitive control sensors, or pharmacological blockade of the GPCR binding site. A separate approach altogether is to measure hemodynamic responses directly, using fluorescence in FP [58] or, in micro- and mesoscopic systems, reflectance [59]. This requires more sophisticated equipment, but in return offers not only control for a significant source of artifacts, but a useful result in its own right.

No single method here is perfect. For instance, not all systems for conducting FP experiments are equipped with the ability to record such a signal (or may not have filters well-suited for a particular sensor), isosbestic points are known for some, but not all, sensors (for some examples, see Table 2 in: [11]), and separation between “true” signal and isosbestic control can be questionable and may require additional assumptions (see below). Expressing a constitutive fluorescent control at the same site, on the other hand, limits the possibility of using several sensors, and should, at a minimum, be strictly checked for between-channel bleedthrough. Other approaches require performing measurements in separate animals, or can be only applied at the end of an experiment – this is the case for mutant sensors and sensor blocking, respectively – so cannot serve as a reference during a recording. Therefore, it is always necessary to test the selected sensor and control choices beforehand, checking the appropriate titer and injection site vs. the signal-to-noise ratio and ability to detect the expected event. For best results, especially for relatively weak signals, it is best to perform several controls to increase confidence in the observed results, and use blind assessment of responses to stimulation or behavior in animals expressing sensor vs. mutant control sensor or constitutive fluorescent protein control, as bias is just human nature and “seeing” spurious patterns in noisy signal is just par for the course.

### Data processing and interpretation

With the rise in popularity of both fiber photometry and fluorometric sensors in general, a wide variety of hardware solutions are readily available. These range from out-of-the-box, plug-and-play commercial products, to open-source “kits” [60, 61], to completely custom-built marvels of ad-hoc engineering. Likewise, for data processing and analysis, a plethora of software tools exists, operating in Python, Matlab, R, LabView, and other environments: this includes proprietary software bundled together with the above-mentioned commercial solutions, as well as completely free and open libraries and programs [61–65]. As is the case with most experimental techniques, the trade-off here is – price notwithstanding – between ease of use, represented by “closed” systems, versus control, capability and customizability at the cost of more demand put on the end user. Data analysis in FP is a swiftly developing field, as is biostatistics in general: there are many sophisticated tools and analytical techniques described in the literature, meriting a systematic review on their own. Here, we will mostly cover the basics required to sensibly use the available ready-to-run tools.

Working with FP data usually entails two major steps: pre-processing of the raw signal and data analysis. Commonly, in the pre-processing step, a digital signal is filtered (with a low-pass filter that should be set to correspond to the characteristics of the signal, such as sampling rate) and normalized to a control value obtained with one of the above-mentioned methods. It is then reported as ΔF/F ($\:\frac{signal\:-\:fitted\:control}{fitted\:control})$, or processed further, often with some variant of z-score normalization. The latter makes sense when comparing values in a single animal between sessions, or between different subjects.

There are two major caveats regarding this normalization step. Firstly, the background is assumed to be uncorrelated to the (specific) sensor signal. This, however, requires at least some initial verification, as the control signal can be “contaminated” by sensor fluorescence, or vice versa. This may potentially result in a slight positive or negative correlation between the control and experimental signal: for instance, if the isosbestic channel captures a portion of the experimental signal (such as during a high-amplitude epoch or transient in the recording), it would be “overcorrected”; conversely, if the control signal is captured by the experimental channel, it would additively affect and potentially even be misidentified as the observed response [11]. For an extended discussion of modeling isosbestic signal with least squares regression, see [66]. Secondly, the normalized value (such as z-score) depends on the reference window in which it is calculated (e.g. from the entire recording, static defined window, moving window of defined length) and, potentially more importantly, on the characteristics of the signal. If the signal has significant artifacts, or simply high-amplitude events, then the denominator of the z-score calculation will be unnecessarily large and the signal of interest will be underestimated. Artifact subtraction before data normalization is, therefore, typically performed (and part of the normal workflow); data processing tools often allow the user to choose the best normalization method (using either the standard deviation or median deviation metric of the signal or of designated “baseline” epochs), sometimes also offering smoothing or robust estimators (e.g. calculating the “baseline” after removing all values above some defined absolute deviation). This means that the user should: (1) familiarize themselves with how the value obtained is calculated, based on the documentation of any algorithm or library being used, (2) test whether the chosen method is reliable in detecting the type of transient or event-signal relation necessitated by the research question during initial exploration and testing of the sensor, before generating full experimental data. The latter is critical for data hygiene, as choosing pre-processing methods *a posteriori* runs the risk of either discovering none of the options capture the recorded data in a satisfactory manner or, worse yet, introducing bias to the results by inadvertently shaping the data to the (expected) answer. While this cannot be truly avoided, steps should be taken for this risk to be at least minimized.

Eventually, the data obtained in the above steps needs to be summarized and analyzed. Most commonly, if any sort of discrete events are present, the simplest and most straightforward visualization method is to create peri-event estimates and visualizations (histograms, density plots) of the signal. At this point, the maximum amplitude of the peaks or area under the curve measurements can be compared with fairly standard hypothesis tests. This simple approach can sometimes be sufficient, although it also has some issues: the answer it provides depends on the window in which the signal was summarized (and therefore, introduces a conundrum: a short window might not fully capture the peri-event activity, a long window says less about the temporal relation between event and signal). Furthermore, it provides no information about the remainder of the signal – such as, for instance, if the transient observed in response to the signal is unique to the event, ubiquitous, or anything in between. For this reason, some authors propose modeling the entirety of the signal and testing for the distinct, but related question “do detected transients arise more often in relation to the event”. Here, non-parametric methods (e.g. permutation, bootstrapping) can help obtain reliable confidence intervals from FP data which are not necessarily normally distributed; for a demonstration of those techniques see: [66, 67].

Another statistical technique readily applicable to FP signal is using linear, or, more commonly, generalized linear models to estimate coefficients related to experimental variables (reward, cue presentation, operant behavior), either categorical or numeric. Here, care must be taken to take into consideration the random nature of signals across individuals and data points (necessitating the use of mixed models), and the usual caveats regarding multiple regression, such as collinearity (correlation between predictors). For an example of this approach, with a practical worked example, see: [11]. In addition, a recently published study [68] has proposed a new flexible mixed model-based framework for FP data analysis that is currently in development and available as a library in the R environment.

The final caveat has less to do with the peculiarities of photometry or statistical modeling in general, and more with assigning biological significance to observed phenomena in experiments combining measurement of brain states and behavioral readout. Avoiding spurious conclusions from data, even if the data has been correctly obtained, processed, and analyzed, requires taking into consideration the broader experimental context. In the case of neuronal activity (and, one can assume, transmitter release events), not all activity *associated* with an observed phenomenon represents activity *functionally relevant* to the observed phenomenon. First observed in mPFC recordings from rats [69, 70] and later dubbed the Euston–Cowen–McNaughton Hassle [71], there exists a tendency for a functional or cognitive function under study to be convolved (and therefore, highly explained) by neuronal activity corresponding to the locomotor component of behavior. At the time of its discovery, this relationship was slightly surprising, but in practical terms, it now represents an important caveat that needs to be taken into account: e.g. by collecting fine-grained visual data about the motor responses of the animal at each trial and modeling them explicitly, or implicitly taking them into account by e.g. selecting trials based on similarity of the motions of the actual animal. This loops back to experimental design.

### Photometry in the near future

- Fluorescent sensors offer a rich repertoire of experimental tools that can be adapted for a variety of research questions and readily combined with other techniques. The entire field is undergoing a rapid development phase, and in the near future, one can expect more available, more sophisticated, and more interconnected methods and equipment. There are several areas of ongoing progress.
- Firstly, quantitative fluorometry; made possible by the development of a wider suite of reliable fast GEVIs and ratiometric sensors based on FRET [72, 73] or advances in sensors optimized for fluorescence time constant response to analyte concentration [74, 75]. Such sensors are promising, as they could, theoretically, be independent of bleaching or local density and amenable to proper calibration and quantification. The equipment used for these measurements is significantly more complex, costly and demanding than using photodetector or camera systems to simply measure fluorescence intensity, but with wider adoption, there is room for future improvement. Advances in hardware in general represent an important step forward for the field: this includes more efficient control over beam shape, allowing for anatomically precise measurements [16], enhanced multiplexing via fabrication of fiber arrays in a similar fashion to electrode arrays [76], and more readily available and robust multi-channel systems, especially in combination with spectrometric measurements leveraging time-correlated single photon counting to obtain fluorescence lifetime and spectral characteristics, in addition to intensity [15, 24]. This technique allows for effective spectral unmixing of co-localized and partly overlapping signals in vivo based on empirical spectra [77].
- Next, the emergence of more advanced analysis and statistical modeling tools, which would include applying some of the statistical techniques mentioned previously (e.g. to achieve robust or event-agnostic modeling of the signal), perhaps also incorporating Granger causality [78] or similar techniques for the study of time-series from multiple sites or wide areas. Many of these mathematical tools exist, but their testing, validation and making them more available for the regular user (such as by incorporation into commonly used software, both open-source and commercial) would certainly empower FP data analysis.
- Beyond the current incremental improvements of fluorescent sensors, different phenomena can be harnessed in the construction of genetically encoded indicators of different types: for instance, in a recent study, a glutamate sensor applying bioluminescence was demonstrated [79]. A red-shifted sensor of this class would allow for much deeper tissue penetration, or potentially intact transcranial imaging, especially in combination with enhanced delivery methods, some of which have had their initial proof-of-concept debut [80].

In summary, one can expect fascinating developments in this space, and it seems highly likely at this point that photometric sensors and methods will rapidly become a prevalent, and standard, scientific tool in a matter of several years, in a fashion similar to previous breakthrough technologies, such as optogenetic manipulation or confocal microscopy.

## Data Availability

No datasets were generated or analysed during the current study.

## Abbreviations

cpGFP: Circularly permuted green fluorescent protein
FP: Fiber photometry
FRET: Förster energy transfer
GECI: Genetically encoded calcium indicator
GEVI: Genetically encoded voltage indicator
GPCR: G protein-coupled receptor
GRAB G: Protein-coupled receptor activation-based sensor
ILN: Thalamic intralaminar nucleus
LHb: Lateral habenula
mPFC: Medial prefrontal cortex
PBP: Periplasmic binding protein
SCN: Suprachiasmatic nucleus
VTA: Ventral tegmental area
